# Embryonic defence mechanisms against glucose-dependent oxidative stress require enhanced expression of *Alx3* to prevent malformations during diabetic pregnancy

**DOI:** 10.1038/s41598-017-00334-1

**Published:** 2017-03-24

**Authors:** Patricia García-Sanz, Mercedes Mirasierra, Rosario Moratalla, Mario Vallejo

**Affiliations:** 1 Instituto de Investigaciones Biomédicas Alberto Sols, Consejo Superior de Investigaciones Científicas (CSIC)/Universidad Autónoma de Madrid, and Centro de Investigación Biomédica en Red de Diabetes y Enfermedades Metabólicas Asociadas CIBERDEM, Madrid, Spain; 2 Instituto Cajal, Consejo Superior de Investigaciones Científicas (CSIC), Madrid, and CIBERNED, Instituto de Salud Carlos III, Madrid, Spain

## Abstract

Oxidative stress constitutes a major cause for increased risk of congenital malformations associated to severe hyperglycaemia during pregnancy. Mutations in the gene encoding the transcription factor ALX3 cause congenital craniofacial and neural tube defects. Since oxidative stress and lack of ALX3 favour excessive embryonic apoptosis, we investigated whether ALX3-deficiency further increases the risk of embryonic damage during gestational hyperglycaemia in mice. We found that congenital malformations associated to ALX3-deficiency are enhanced in diabetic pregnancies. Increased expression of genes encoding oxidative stress-scavenging enzymes in embryos from diabetic mothers was blunted in the absence of ALX3, leading to increased oxidative stress. Levels of ALX3 increased in response to glucose, but ALX3 did not activate oxidative stress defence genes directly. Instead, ALX3 stimulated the transcription of *Foxo1*, a master regulator of oxidative stress-scavenging genes, by binding to a newly identified binding site located in the *Foxo1* promoter. Our data identify ALX3 as an important component of the defence mechanisms against the occurrence of developmental malformations during diabetic gestations, stimulating the expression of oxidative stress-scavenging genes in a glucose-dependent manner via *Foxo1* activation. Thus, ALX3 deficiency provides a novel molecular mechanism for developmental defects arising from maternal hyperglycaemia.

## Introduction

Maternal pre-gestational diabetes is a major cause for increased risk of embryonic damage and congenital malformations during pregnancy^1, 2^. Malformations caused by diabetic embryopathy can affect any tissue, but the most frequent involve the neural system and the heart^3, 4^. In humans it has been difficult to establish that embryonic malformations are solely a direct effect of glucose toxicity due to the complex metabolic alterations that are characteristic of diabetes mellitus. However, animal studies indicate that exposure of developing embryos to high glucose levels is sufficient to cause cellular damage leading to severe malformations^5, 6^.

Congenital neural tube closure and heart defects are frequently observed in mouse or rat embryos developing during diabetic pregnancies^5–10^. Animal models and clinical studies have identified a relatively large number of genes associated to these malformations^11, 12^. However, the mechanisms by which maternal diabetes increases the vulnerability of embryos to malformations in humans remain largely elusive.

Many of the embryonic malformations arising as a consequence of gene inactivation or of diabetic gestations usually appear with incomplete penetrance^10, 12–16^, indicating the possible existence of genetic predisposition^17–19^ or the influence of environmental factors^15, 19, 20^. A major contributor to embryonic malformations associated to maternal hyperglycaemia is the increased production of reactive oxygen species (ROS) leading to oxidative stress^21–23^, accompanied by decreased ability of cells to activate antioxidant defence mechanisms^24–27^. Oxidative stress may contribute considerably to excessive apoptosis during development^28, 29^, which in turn constitutes an important determinant for the appearance of malformations in developing embryos^5, 29^.

The gene encoding the homeodomain transcription factor ALX3 is expressed in mesenchymal cells in mouse mid gestation embryos and regulates important developmental processes^30–33^. Recessive mutations in this gene in humans or its deficiency in mice result in craniofacial malformations as well as in cranial neural tube defects accompanied by increased apoptosis in the forehead mesenchyme^32, 34^. In mice, this phenotype exhibits a characteristic incomplete penetrance so that a considerable proportion of *Alx3*-deficient embryos are apparently normal and viable^32, 33^. In adult mice, *Alx3* is expressed in pancreatic islets, and its deficiency leads to apoptosis of islet cells and alterations in glucose homeostasis without reaching overt diabetes^35, 36^. Based on these observations and on the association between *Alx3*-deficiency and apoptosis^32, 35^, we investigated whether *Alx3*-deficient embryos are more vulnerable to the harmful effects of hyperglycaemia during diabetic pregnancy.

## Results

### Generation of diabetic pregnancies

**Figure 1:**
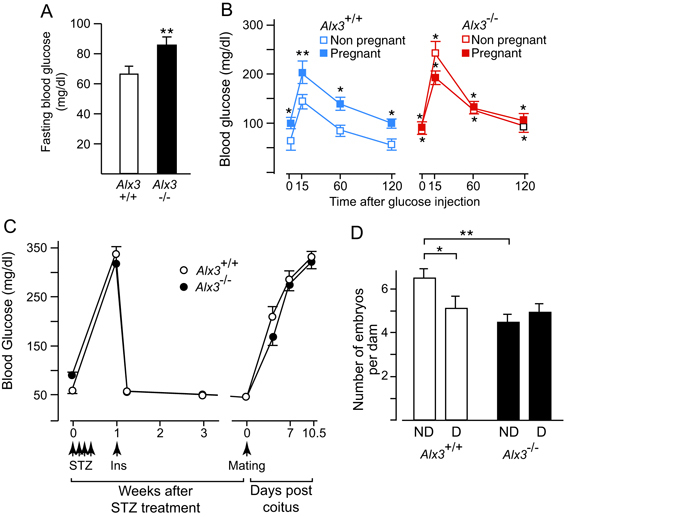
Induction of diabetic pregnancies in wild type and *Alx3*-deficient mice. (**A**) Basal blood glucose concentrations after fasting in wild-type (n = 27) and *Alx3*-deficient (n = 38) female mice. **P < 0.01, Student’s t-test. (**B**) Glucose tolerance tests carried out in fasting wild-type (left panel, blue, n = 21) or *Alx3*-deficient (right panel, red, n = 19) female mice. Both non-pregnant (clear squares) and pregnant mice (solid squares) at 8.5 days post coitus were tested. *P < 0.05, **P < 0.01 relative to non pregnant wild type animals at each time point (ANOVA followed by Bonferroni transformation). No statistically significant differences were found between pregnant and non-pregnant *Alx3*-null females. (**C**) Blood glucose concentrations after fasting in wild type (white circles, n = 22) and *Alx3*-deficient (black circles, n = 43) female mice. Injections of streptozotocin (STZ) and subcutaneous implantation of insulin pellets (Ins) are indicated by arrows. Note that severe hyperglycaemia develops again during pregnancy up to 10.5 days of gestation. (**D**) Number of embryos per litter observed in non-diabetic (ND) or diabetic (D) pregnancies at 10.5 days of gestation. The numbers of litters were: *Alx3* ^+/+^, 20 (ND) and 15 (D); *Alx3* ^−/−^, 19 (ND) and 29 (D). Pregnancies without any embryos were not counted for litter size calculations. *p < 0.05; **P < 0.01, Student’s t-test. All values represent mean $\mathop{+}\limits_{-}$ s.e.m.

In line with our previous findings^35^, fasting blood glucose levels were higher in non-pregnant *Alx3*-deficient than in wild type females, but they remained with normal range (<120 mg/dl) and never reached levels characteristic of overt diabetes, defined as fasting blood glucose concentrations higher than 250 mg/dl (Fig. 1A). Since non-pregnant *Alx3*-deficient females had only a mild hyperglycaemia, we sought to determine whether pregnancy in these animals was associated with increased basal blood glucose levels or severe glucose intolerance. Glucose tolerance tests demonstrated the presence of relatively mild glucose intolerance in *Alx3*-deficient non-pregnant females as compared with wild type non-pregnant controls (Fig. 1B). When tests were carried out in pregnant females, we found that wild type animals had developed a degree of glucose intolerance similar to that found in *Alx3*-deficient females (Fig. 1B), consistent with the normal metabolic adaptations of maternal glucose homeostasis during pregnancy^37–39^. In contrast, pregnancy did not aggravate further the glucose intolerance observed in *Alx3*-deficient females, which remained similar to the glucose intolerance observed in wild type pregnant females (Fig. 1B). In addition, no significant differences were found in serum insulin levels of pregnant or non-pregnant *Alx3*-deficient females as compared to those found in wild type animals (Supplementary Table S1). All these data indicate that *Alx3*-deficient non-pregnant females have mild glucose intolerance, but this is not aggravated during pregnancy, being similar to the glucose intolerance associated with pregnancy in wild type animals. Therefore, this mild alteration of glucose homeostasis cannot be expected to cause embryonic malformations.

Since *Alx3*-deficient mice do not develop overt diabetes upon pregnancy, we induced diabetic gestations by treating female mice with streptozotocin to provoke the selective destruction of pancreatic islets, a widely accepted and commonly used method for the study of diabetic embryopathy^5–10, 13, 16, 20, 23, 40–43^. Pancreatic islet destruction by streptozotocin was reflected by the appearance of hyperglycaemia, which was corrected by subcutaneous implantation of insulin pellets (Fig. 1C). Prior to mating, all streptozotocin-treated female mice implanted with insulin pellets were checked to have similar fasting glucose levels within normal values, indicating the effectiveness of insulin released from the pellets (Fig. 1C). After mating, blood glucose levels increased progressively as a consequence of pregnancy. One week after the onset of gestation they reached values higher than 250 mg/dl, and continued to increase until embryos were extracted for analyses after 10.5 days of gestation (Fig. 1C). This severe hyperglycaemia was not due to exhaustion of the insulin content of the pellets, because streptozotocin-treated non-pregnant females did not develop hyperglycaemia after insulin pellet implantation during a similar period of time.

The average litter size in wild type mice was reduced in the diabetic group relative to the non-diabetic control (Fig. 1D). In the case of *Alx3*-null animals, the number of embryos in non-diabetic pregnancies was lower than in non-diabetic wild type pregnancies, but litter size was not reduced further as a consequence of diabetes (Fig. 1D). In the diabetic mice, the differences in genotype did not affect the rate of empty decidua or the percentage of embryonic resorptions (Table 1).

**Table 1:** Pregnancy outcome in diabetic mice.

Genotype	Number of dams	Percentage of females with empty decidua	Number of implantations	Percentage of resorptions
*Alx3* ^+/+^	22	31.8 (n = 7)	87	10.34 (n = 9)
*Alx3* ^−/−^	43	27.9 (n = 12)	172	11.62 (n = 20)
*Alx3* ^+/−^	44	25 (n = 11)	191	10.47 (n = 20)

### Increased craniofacial malformations in *Alx3*-mutant embryos from diabetic pregnancies

We broadly identified two morphologically distinct types of embryonic malformations. On the one hand, malformations typically observed in *Alx3*-deficient mutants^32^, affecting the craniofacial mesenchyme and the forehead, or defects of the cranial neural tube at the prosencephalic and/or mesencephalic levels. These are referred to in the present study as craniofacial malformations. On the other hand, malformations consisting mostly of tail flexion defects similar to those defined by Copp *et al.* ^44^, occasionally including dorsal neural tube closure defects at the rhombencephalic or spinal levels. These are referred to in this study as caudal malformations.

No malformations were observed in wild type embryos from non-diabetic pregnancies (Table 2). Consistent with previous observations^32^, a relatively small proportion of *Alx3* ^+/−^ and *Alx3* ^−/−^ embryos developing in non-diabetic mothers showed cranial malformations affecting the closure of the cranial neural tube (Table 2 and Supplementary Fig. S1).

**Table 2:** Number of malformed or non-malformed embryos at 10.5 days of gestation during non-diabetic or diabetic pregnancies.

Genotype	Diabetic mother	Number of embryos examined	Number of non-malformed embryos	Number of embryos with craniofacial malformations	Number of embryos with caudal malformations	Total percentage of malformed embryos
*Alx3* ^+/+^	No	132	132	0 (0)	0 (0)	0
*Alx3* ^+/−^	No	192	188	4 (2.08)	0 (0)	2.08
*Alx3* ^−/−^	No	86	82	4 (4.65)	0 (0)	4.65
*Alx3* ^+/+^	Yes	78	74	0 (0)	4 (5.12)	5.12
*Alx3* ^+/−^	Yes	171	147	13 (7.6)^a,d^	11 (6.43)	14.03^f^
*Alx3* ^−/−^	Yes	152	115	24 (15.78)^b,c,e^	13 (8.55)	24.34^g^

Numbers in parenthesis denote percentage of total.

^a^ *p* < 0.05 relative to *Alx3* ^+/−^ embryos from non-diabetic mothers; Fisher’s exact test.

^b^ *p* < 0.05 relative to *Alx3* ^+/−^ embryos from diabetic mothers; Fisher’s exact test.

^c^ *p* < 0.05 relative to *Alx3* ^−/−^ embryos from non-diabetic mothers; Fisher’s exact test.

^d,f^ *p* < 0.001 relative to *Alx3* ^+/−^ embryos from non-diabetic mothers plus *Alx3* ^+/+^ embryos from diabetic mothers; χ^2^ test.

^e,g^ *p* < 0.001 relative to *Alx3* ^−/−^ embryos from non-diabetic mothers plus *Alx3* ^+/+^ embryos from diabetic mothers; χ^2^ test.

**Figure 2:**
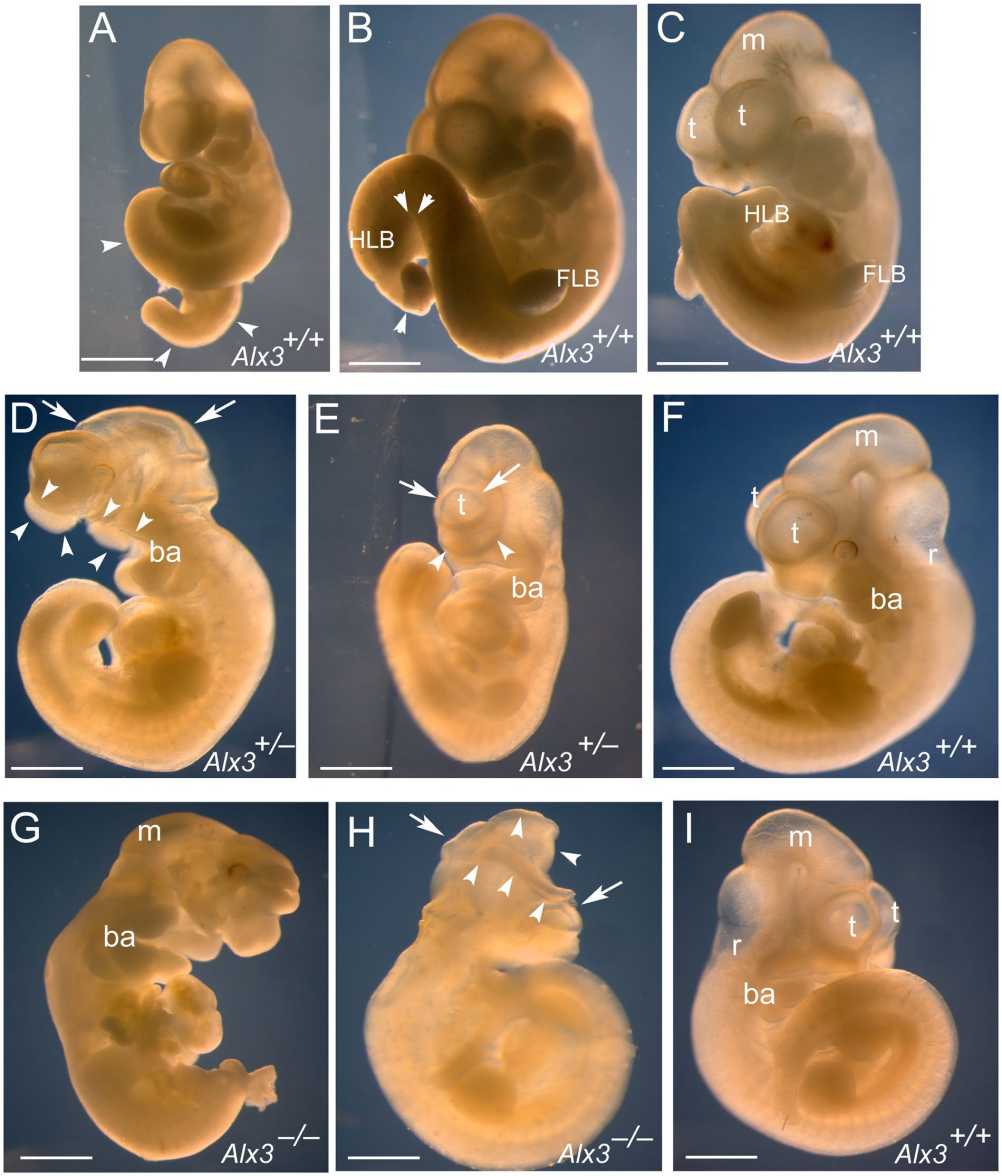
Malformations observed in embryos from diabetic pregnancies after 10.5 days of gestation. (**A,B**) Examples of caudal malformations observed in wild type embryos from diabetic pregnancies. Tail flexion defects are indicated by arrowheads. (**D**,**E**) Examples of cranial malformations observed in heterozygote *Alx3*-deficient embryos from diabetic pregnancies. These include defects of the rostral mesenchyme affecting midline closure at the level of the forehead (**D**, arrowheads), defects at the level of the mesencephalic vesicles (**D**, arrows) and defects of facial mesenchyme expansion (**E**, arrowheads) affecting the development of the telencephalic vesicles (**E**, arrows). (**G,H**) Examples of severe malformations found in *Alx3*-null embryos developing in diabetic mothers. Malformations affected several embryonic regions including the forehead mesenchyme, the branchial arches and the cranial neural tube (**G**), or were restricted to severe craniofacial and cranial neural tube closure defects (**H**, arrows). Arrowheads indicate open neural folds. (**C**,**F** and **I**) depict normal wild type embryos for comparisons. Scale bars represent 1 mm in all cases except in **G** (1.5 mm). Abbreviations: ba, Branchial arches; FLB, Forelimb bud; HLB, Hind limb bud; m, Mesencephalon; r, Rhombencephalon; t, Telencephalic vesicles.

In diabetic gestations, a proportion of wild type embryos exhibited caudal malformations consisting of tail flexion defects (Table 2 and Fig. 2A,B), but no cranial malformations were observed. In the case of *Alx3* ^+/−^ embryos, maternal diabetes increased the number of embryos with craniofacial malformations (Table 2, superscript a; and Fig. 2D and E). We also found caudal malformations affecting the tail in a similar proportion to those found in wild type embryos from diabetic mothers (Table 2). The incidence of craniofacial malformations found in Alx3^+/−^ embryos was not significantly affected by the genotype of the dams: 6.77% (n = 59) from crossings between wild type females and Alx3^−/−^ males, and 8.03% (n = 112) from crossings between Alx3^−/−^ females and wild type males.

*Alx3* ^−/−^ embryos from diabetic pregnancies showed an even higher proportion of craniofacial malformations (Table 2, superscript b). These malformations were considerably more severe than those observed in *Alx3* ^+/−^ embryos, generating clearly dysmorphic embryos (Fig. 2G,H). In contrast, the proportion of caudal malformations was not significantly different from those found in heterozygote or wild type embryos from diabetic mothers (Table 2). When compared with non-diabetic pregnancies, maternal diabetes significantly increased the number of *Alx3* ^−/−^ embryos with severe craniofacial malformations (Table 2, superscript c). Remarkably, the proportion of craniofacial malformations in *Alx3* ^+/−^ or *Alx3* ^−/−^ embryos from diabetic pregnancies was considerably higher than expected if the effects of diabetes and *Alx3* deficiency were additive (Table 2, superscripts d and e, respectively). A similar result was obtained when the proportion of total malformations (craniofacial and caudal together) was considered for each genotype (Table 2, superscripts f and g). In summary, craniofacial malformations due to *Alx3* inactivation or haploinsufficiency increase considerably in diabetic gestations. This synergistic increase indicates that *Alx3* deficiency renders the embryos more vulnerable to the teratogenic effects of hyperglycaemia.

### Increased oxidative stress in *Alx3*-deficient embryos from diabetic pregnancies

**Figure 3:**
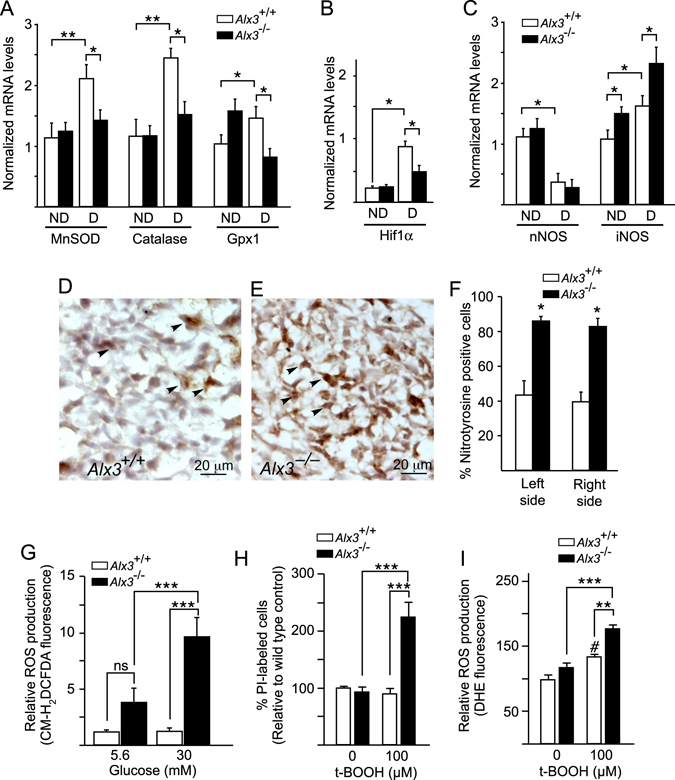
Impaired activation of genes encoding oxidative stress-scavenging enzymes and increased oxidative stress in *Alx3*-deficient embryos developing during diabetic pregnancies. (**A**–**C**) Relative levels of mRNA extracted from embryos of non-diabetic (ND) or diabetic (D) wild type (white bars) or *Alx3*-deficient (black bars) mice, as assessed by quantitative RT-PCR after 10.5 days of gestation. Shown are mRNAs encoding manganese superoxide dismutase (MnSOD), catalase and glutathione peroxidase-1 (Gpx1) (**A**), Hif1α (**B**) or neuronal (n) or inducible (i) nitric oxide synthase (NOS) (**C**). *P < 0.05; **P < 0.01; Student’s t-test (n = 7 per group). (**D** and **E**) Representative sections showing nitrotyrosine immunohistochemistry in the head mesenchyme of *Alx3* ^+/+^ (**D**) or *Alx3* ^−/−^ (**E**) embryos from diabetic pregnancies. Arrowheads indicate representative examples of nitrotyrosine-positive cells. (**F**) Quantification of the percentage of nitrotyrosine-positive cells detected by immunohistochemistry in sections from wild type (white columns) or *Alx3*-deficient (black columns) embryos from diabetic pregnancies. Equivalent mesenchyme regions symmetrically located at both left and right sides of each section relative to the midline of the embryo were scored independently. Approximately 1000 cells per section from 6 sections obtained from 3 embryos in each group were counted. *P < 0.001, Student’s t-test. (**G**) Quantitation of ROS in control (white bars) or *Alx3*-deficient (black bars) primary MEM cells cultured in the presence of the indicated concentrations of glucose and labelled with the fluorescent dye CM-H_2_DCFDA (n = 6 per group). (**H** and **I**) Response of control or *Alx3*-deficient primary MEM cells to t-BOOH treatment. In **H**, the relative numbers of non-viable cells identified by propidium iodide (PI) staining is represented (n = 14 for untreated cell groups; n = 12 for t-BOOH-treated cell groups). In **I**, ROS production as measured by DHE fluorescence is shown (n = 7 for untreated cell groups; n = 6 for t-BOOH-treated cell groups). In (**G**–**I**), *P < 0.05; **P < 0.01; ***P < 0.001; ^#^ *P* < 0.05, compared with untreated wild type cells. ANOVA followed by Bonferroni test. All values represent mean $\mathop{+}\limits_{-}$ s.e.m.

Since high glucose levels favour the generation of ROS in the embryos, we argued that the increase in cranial malformations observed in *Alx3*-deficient embryos from diabetic mothers could reflect a role for ALX3 in the activation of defence mechanisms against oxidative stress. To test this hypothesis, we determined whether lack of ALX3 could affect the expression levels of genes that encode oxidative stress-scavenging enzymes. The expression of manganese superoxide dismutase (MnSOD), catalase, and glutathione peroxidase-1 (Gpx1) in *Alx3*-deficient embryos from non-diabetic mothers were similar to those observed in non-diabetic wild type embryos (Fig. 3A). Maternal diabetes increased the expression of these genes in wild type embryos but not in *Alx3*-deficient embryos (Fig. 3A). Expression of *Hif1α*, also involved in the defence response to oxidative stress^41, 42^, was similar in wild type and *Alx3*-deficient embryos from non-diabetic mothers (Fig. 3B). In this case, maternal diabetes resulted in increased expression in wild type but not in *Alx3*-deficient embryos (Fig. 3B).

In addition, expression of neuronal nitric oxygen synthase (NOS) was lower in embryos from diabetic pregnancies than in those from non-diabetic pregnancies, but in each of these conditions we found no significant differences between wild type and *Alx3*-deficient embryos (Fig. 3C). In contrast, maternal diabetes increased the expression of inducible NOS, but this effect was higher in *Alx3*-deficient than in wild type embryos (Fig. 3C). Consistent with these data, we detected increased nitrotyrosine staining in the head mesenchyme of *Alx3*-null embryos from diabetic pregnancies as compared to wild type embryos (Fig. 3D–F). These results suggest that the defence response against oxidative stress in the presence of high glucose levels is impaired in *Alx3*-deficient embryos.

To directly test whether *Alx3*-deficiency is accompanied by increased oxidative stress, we prepared primary mouse embryonic mesenchyme (MEM) cells directly from wild type or *Alx3* knockout embryos. These cells normally express *Alx3* abundantly^30, 32^ and are located in the craniofacial region where malformations in *Alx3*-deficient embryos were found. Using these primary MEM cells we measured ROS production after exposure to low or high concentrations of glucose using fluorescent probes. When cultured in the presence of 5.6 mM glucose, a concentration corresponding approximately to euglycaemic levels *in vivo*, ROS production levels in cells from *Alx3*-deficient embryos were not significantly different from those found in control cells from wild type embryos (Fig. 3G). Increasing the concentration of glucose to 30 mM resulted in a significant elevation in the intracellular levels of ROS only in *Alx3*-deficient cells (Fig. 3G).

Then we asked whether lack of ALX3 increases the vulnerability of cells to death by oxidative stress. Initially, using DAPI, we determined that exposure to a relatively high concentration of the oxidative stress-inducing agent tert-butyl-hydroperoxide (t-BOOH; 300 μM) resulted in a significant increase in the number of non viable primary MEM cells prepared from wild type embryos (Supplementary Fig. S2). Notably, the proportion of non-viable MEM cells prepared from *Alx3*-deficent embryos was significantly higher compared to that of wild type cells (Supplementary Fig. S2). To confirm these results, we treated MEM cells with a sub-lethal concentration of t-BOOH (100 μM) and determined the proportion of cell death in wild type and *Alx3*-deficient primary MEM cells using propidium iodide. In this case, exposure to t-BOOH only resulted in significantly increased death in the case of cells derived from *Alx3*-deficient embryos, whereas cells from wild type embryos were unaffected (Fig. 3H and Supplementary Fig. S2). These experiments indicate that embryonic mesenchyme cells are more vulnerable to oxidative stress in the absence of ALX3.

Incubation of cells with DHE indicated that superoxide production did not contribute significantly to the elevated ROS pool in *Alx3*-mutant cells (Fig. 3I). Nonetheless, exposure of cells to t-BOOH (100 μM) generated significantly higher levels of the superoxide anion in *Alx3*-deficient than in wild type MEM cells (Fig. 3I and Supplementary Fig. S2).

### ALX3 regulates the expression of *Foxo1*

**Figure 4:**
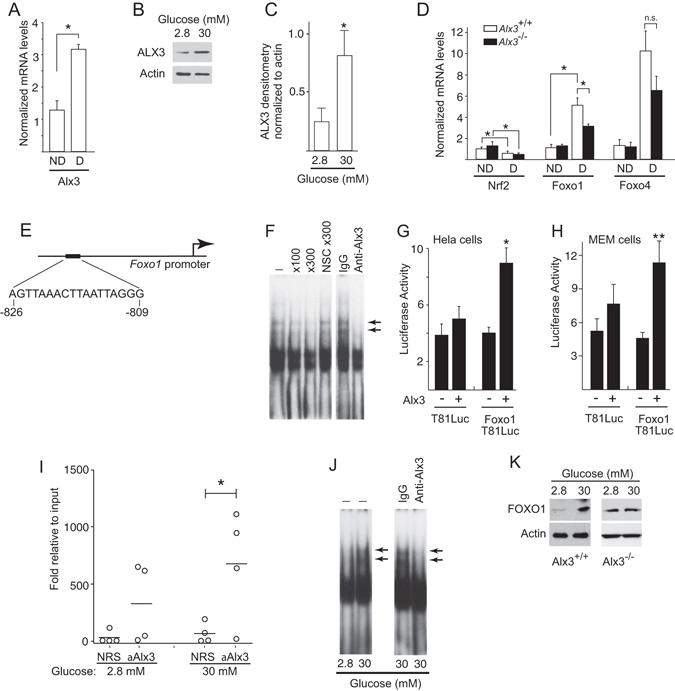
Regulation of the *Foxo1* promoter by ALX3. (**A**) Quantitative RT-PCR of *Alx3* mRNA in embryos from non-diabetic (ND) or diabetic (D) wild type mice (n = 7 per group). (**B**) Western blot showing ALX3 in primary MEM cells cultured in the indicated concentrations of glucose. (**C**) Densitometric measurements of ALX3 bands relative to actin bands from three western blots similar to that shown in **B**. **D**) Quantitative RT-PCR of *Nrf2*, *Foxo1* or *Foxo4* mRNAs, extracted from embryos of non-diabetic (ND) or diabetic (D) wild type (white bars) or *Alx3*-deficient (black bars) mice; n.s., non-significant (n = 7 per group). (**E**) ALX3-binding site and its position in the mouse *Foxo1* promoter. (**F**) Electrophoretic mobility shift assays showing the binding of proteins from nuclear extracts of primary MEM cells to an oligonucleotide probe containing the sequence indicated in **E**. The absence (−) or presence of competing or nonspecific competing (NSC) oligonucleotides at the indicated fold molar excess, or of an ALX3 antibody or control IgG, is depicted on top. Arrows indicate complexes containing ALX3. (G,H) Relative luciferase activity elicited in Hela (**G**) or primary *Alx3*-defcient MEM (**H**) cells co-transfected with the reporter plasmids Foxo1T81Luc or control pT81Luc, and an ALX3 expression plasmid (n = 5). (**I**) Quantitative PCR amplification of *Foxo1* chromatin immunoprecipitated with anti-ALX3 antiserum or with control non-immune rabbit serum (NRS) from primary MEM cells isolated from wild type mice and cultured in the presence of the indicated concentrations of glucose. Horizontal lines represent the mean (n = 4). (**J**) Electrophoretic mobility shift assays showing binding of nuclear extracts from primary MEM cells cultured at the indicated concentrations of glucose to the *Foxo1* promoter site indicated in **E**. Arrows indicate the presence of ALX3. (**K**) Western blot showing FOXO1 in primary wild type (left panels) or *Alx3*-deficient (right panel) MEM cells cultured in the indicated concentrations of glucose. A representative example of three independent experiments with similar results is depicted. Uncropped blots are provided in Supplementary Fig. S5. Except in **I**, all values represent mean $\mathop{+}\limits_{-}$ s.e.m. *P < 0.05, **P < 0.01, Student’s t-test.

Impaired expression of defence genes against oxidative stress leading to excess ROS production in *Alx3*-deficient embryos under hyperglycaemic conditions could reflect a possible role for ALX3 in the regulation of the transcriptional transactivation of these genes. Consistent with this hypothesis, we found that maternal diabetes results in increased expression of *Alx3* mRNA in wild type embryos (Fig. 4A), and that the levels of ALX3 protein were elevated when primary MEM cells were cultured in the presence of high rather than low glucose (Fig. 4B,C), indicating that *Alx3* is a glucose-responsive gene in mouse embryos. However, *in silico* analysis (http://www.dcode.org) of 10 kb regions of the promoters of the genes encoding MnSOD, catalase, Gpx1, and Hif1*α* did not predict the existence of binding sites for ALX3, characterized by the existence of a consensus TAAT motif^45^. We then argued that ALX3 could regulate the expression of these genes indirectly, by stimulating the expression of key transcription factors known to activate the oxidative stress defence response such as *Nrf2* ^46^, *Foxo1* and *Foxo4* ^16, 47, 48^.

Maternal diabetes reduced the expression of *Nrf2* to a similar degree in wild type and *Alx3*-null embryos, making it an unlikely candidate as a target for regulation by ALX3 (Fig. 4D). On the contrary, maternal diabetes increased the expression of *Foxo1*, but this response was significantly blunted in *Alx3*-null embryos (Fig. 4D). Expression of *Foxo4* was also increased in embryos developing in diabetic pregnancies, but the difference between *Alx3*-deficient and wild type embryos was not statistically significant (Fig. 4D). Thus, these experiments indicate that lack of ALX3 impairs induced expression of *Foxo1* in diabetic pregnancies, and suggested that this gene could be a direct target for regulation by ALX3.

Using *in silico* analysis, we identified a potential target site for binding by ALX3 in the *Foxo1* promoter located between nucleotides –826 to –809 relative to the transcription initiation site (Fig. 4E). Therefore, we investigated whether ALX3 regulates the expression of *Foxo1* directly. Direct binding of ALX3 to the target *Foxo1* promoter site was confirmed by electrophoretic mobility shift assays using nuclear extracts from primary MEM cells from wild type embryos. Sequence specificity of DNA–protein complexes was determined by competition with unlabelled oligonucleotides added in excess to the binding reaction, and the presence of ALX3 in these complexes was confirmed by the addition of an ALX3 antibody (Fig. 4F). Using transfections of a luciferase reporter under the control of the *Foxo1* promoter in Hela or in primary MEM cells from *Alx3*-null embryos, we found that overexpression of *Alx3* resulted in increased luciferase activity only when the target site in the *Foxo1* promoter was present (Fig. 4G and H), thus supporting the notion that ALX3 transactivates the *Foxo1* promoter by binding specifically to this site.

Chromatin immunoprecipitation assays using primary MEM cells from wild type embryos demonstrated that ALX3 binds to this region of the endogenous *Foxo1* gene in the context of native chromatin *in vivo* when cells are incubated in high glucose (30 mM), but not in the presence of low glucose (2.8 mM) (Fig. 4I). Using electrophoretic mobility shift assays, binding of ALX3 to the target site of the *Foxo1* promoter was only detected when primary MEM cells were cultured in high rather than low glucose concentrations (Fig. 4J). Consistent with these findings, glucose induced an increase in the levels of FOXO1 protein in cells from wild type embryos, but not in cells from *Alx3*-null embryos (Fig. 4E).

The existence of a possible functional relationship between increased *Alx3* and *Foxo1* levels in the context of abnormally elevated concentrations of glucose was further investigated in primary MEM cells. We argued that if glucose-induced ALX3 is responsible for the increased expression of *Foxo1*, it could be expected that in cells lacking ALX3, expression of *Foxo1* would not occur in response to increased concentrations of glucose. In turn, this would lead to impaired glucose-dependent expression of genes encoding oxidative stress detoxifying enzymes. To test this hypothesis, primary MEM cells were prepared from *Alx3*-null or control wild type embryos from non-diabetic pregnancies.

**Figure 5:**
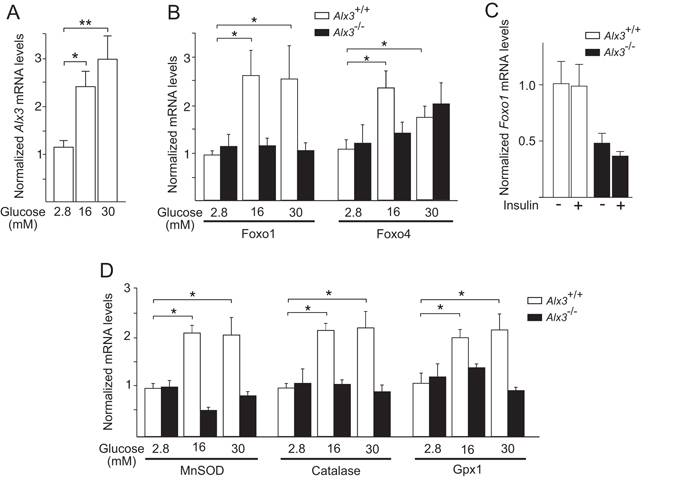
Impaired response to glucose in *Alx3*-deficient cells. (**A**) Relative levels of *Alx3* mRNA in primary MEM cells obtained from wild type mice cultured in the presence of the indicated concentrations of glucose (n = 5). (**B**) Relative levels of *Foxo1* and *Foxo4* mRNA in primary MEM cells obtained from wild type (white bars) or *Alx3*-deficient (black bars) embryos cultured in the presence of the indicated glucose concentrations (n = 7 per group). (**C**) Relative levels of *Foxo1* mRNA in primary MEM cells from wild type (white bars) or *Alx3*-deficient (black bars) embryos cultured in the presence of 0.5% FBS without (−) or with (+) insulin (100 nM) (n = 5 per group). (**D**) Relative levels of MnSOD, catalase and *Gpx1* mRNA in primary MEM cells from wild type (white bars) or *Alx3*-deficient (black bars) embryos cultured in the indicated glucose concentrations (n = 7 per group). All data obtained by quantitative RT-PCR. In all cases, values represent mean $\mathop{+}\limits_{-}$ s.e.m. *p < 0.05, **p < 0.01, Student’s t-test.

Glucose induced the expression of *Alx3* in wild type primary MEM cells (Fig. 5A), confirming our initial results in embryos. Glucose also induced the expression of *Foxo1* and *Foxo4* in these cells (Fig. 5B). In contrast, in cells from *Alx3*-deficient embryos the induction of *Foxo1* expression by glucose was inhibited, whereas that of *Foxo4* was unaffected (Fig. 5B). In addition, in serum starved conditions expression of *Foxo1* was significantly decreased in *Alx3*-deficient MEM cells, but addition of insulin did not result in significant modification of *Foxo1* expression regardless of whether ALX3 was present or not (Fig. 5C). Thus, these experiments indicate that ALX3 regulates the *Foxo1* gene and is specifically required for glucose induced *Foxo1* expression.

Furthermore, expression of mRNAs encoding MnSOD, catalase and Gpx1 was induced by glucose in MEM cells derived from wild type embryos, but not in those derived from *Alx3*-deficient embryos (Fig. 5D). Taken together, these experiments support the notion that high glucose concentrations induce the expression of *Alx3*, which in turn stimulates expression of *Foxo1*, and this subsequently that of genes encoding oxidative stress detoxifying enzymes.

### Altered developmental gene expression in *Alx3*-deficient embryos from diabetic pregnancies

**Figure 6:**
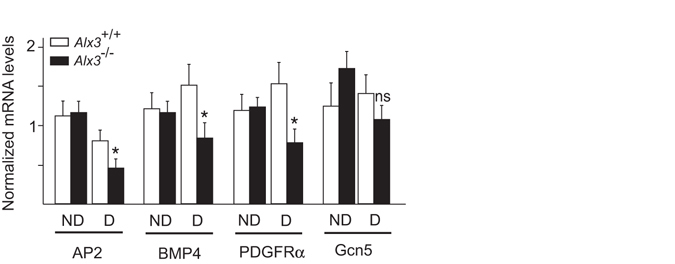
Altered gene expression in *Alx3*-deficient embryos from diabetic pregnancies. Relative levels of mRNA encoding representative developmentally regulated genes in wild type (white bars) or *Alx3*-null (black bars) embryos obtained from non-diabetic (ND) or diabetic (D) mothers. Values represent mean $\mathop{+}\limits_{-}$ s.e.m. *p < 0.05, Student’s t-test (n = 7 per group).

Finally, we investigated whether the alterations observed in *Alx3*-deficient embryos from diabetic pregnancies correlated with altered expression of genes controlling embryonic programs for mesenchyme development. We chose representative examples of genes whose deficiencies have been documented to be associated with developmental defects with partial penetrance. We found that in diabetic pregnancies, expression of *Ap2* ^49^, *Bmp4* ^50^ and *Pdgfrα* ^51^ was reduced in *Alx3*-deficient embryos as compared with wild type embryos (Fig. 6). In contrast, we did not find significant changes in the expression of *Gcn5*, which encodes a histone acetyltransferase not involved in mesenchyme development^52, 53^. No differences in the expression of these genes were found between wild type and *Alx3*-null embryos from non-diabetic pregnancies. Thus, these results show that impaired expression of some developmental genes only appears when both *Alx3*-deficiency and diabetes are present.

## Discussion

Our work indicates that *Alx3* confers protection against oxidative stress produced by glucose in embryonic cells, thus contributing to the prevention of the teratogenic effects of hyperglycaemia during diabetic pregnancy. Glucose up-regulates the expression of *Alx3*, which in turn stimulates the expression of *Foxo1* and the expression of genes encoding oxidative stress-scavenging enzymes as alfa defence mechanism against excess ROS generated by hyperglycaemia. This notion is consistent with the observation of activated expression of oxidative stress-scavenging genes in wild type but not in *Alx3*-deficient embryos developing in diabetic mothers. Although we cannot rule out the existence of epigenetic changes on the target promoters, our data strongly support the notion that *Foxo1* activation in response to glucose is due to increased expression of ALX3 stimulated by glucose itself, and not to a change in the binding efficiency of this transcription factor. That is, in the presence of low concentrations of glucose, ALX3 levels are low, and therefore *Foxo1* promoter occupancy at the specific binding site described in this study is low, thus leading to reduced transactivation. On the contrary, in the presence of high concentrations of glucose, ALX3 levels a high, favoring binding to the *Foxo1* promoter and increasing transactivation.

The mechanism by which glucose stimulates the expression of *Alx3* is unknown, but it appears not to be specific of embryonic cells. In the adult organism, expression of *Alx3* in glucagon-producing cells of the pancreatic islets is also stimulated by glucose. In that case, increased levels of ALX3 in response to elevated glucose result in transcription factor interactions that lead to down-regulation of the glucagon gene, closing a negative feed back loop important for the maintenance of glucose homeostasis^36^. Thus, *Alx3* emerges as an important glucose sensor to protect the organism against the harmful effects of hyperglycaemia. In the present study, *Alx3* deficiency in mouse embryos developing in diabetic mothers resulted in increased oxidative stress, rendering them more vulnerable to congenital malformations.

The increase in craniofacial but not caudal malformations observed in *Alx3*-deficient embryos developing in diabetic pregnancies is in good agreement with the strong expression of *Alx3* in the forehead mesenchyme of wild type embryos^30, 32^, and indicate that in the absence of *Alx3* the mesenchyme cells from this region become vulnerable to the harmfull effects of high glucose concentrations. In line with this reasoning, the most severe malformations observed in *Alx3* ^−/−^ embryos from diabetic mothers could reflect, in addition, increased vulnerability of cells located in other tissues in which *Alx3* is normally expressed, such as the lateral mesoderm or the serous membranes that separate internal organs^30, 32^. Thus, the significant increase in cranial malformations observed when *Alx3* deficiency and diabetes are present together reflects the absence of a protective role for *Alx3* against the teratogenic effects of glucose.

Numerous studies have established a link between oxidative stress and diabetic embryopathy^7, 21, 22, 54^. In *Alx3*-deficient embryonic cells, excess oxidative stress is a consequence of increased production of ROS, as well as of reactive nitrogen species evidenced by elevated nitrotyrosine content and expression of inducible NOS, which has been implicated in the pathogenesis of embryonic malformations induced by maternal diabetes^43, 55^.

Stimulation of the expression of genes encoding oxidative stress-scavenging enzymes has been proposed to act as a mechanism of defence against damage induced by ROS during diabetic pregnancy^26, 27^. Our data indicate that *Alx3* is important for this mechanism of defence for three reasons. First, elevations in glucose concentrations stimulate the expression of *Alx3*, which therefore serves as a glucose sensor to initiate a transcriptional program of defence. Second, glucose-induced stimulation of *Alx3* expression is required for the stimulation of oxidative stress-scavenging genes. And third, *Alx3* is required for the glucose-induced expression of *Foxo1*, a well-known stimulator of oxidative stress-scavenging gene expression during the antioxidant defence response^16, 47, 48, 56, 57^.

We propose that the ALX3-dependent stimulation of the oxidative stress defence response is mediated by an indirect transcriptional activation pathway involving *Foxo1*, because we did not find DNA sequence motifs corresponding to putative ALX3 binding sites in the promoter regions of any of the target genes examined. Taken together, all these data support the proposal that high glucose concentrations favouring the generation of oxidative stress stimulate the expression of *Alx3*, which in turn activates the expression of *Foxo1*, thus stimulating the expression of the genes encoding ROS scavenging enzymes^47, 48^.

The observation that maternal diabetes induced the expression of *Hif1α* in wild type but not in *Alx3*-deficient embryos is in agreement with studies showing an increase in *Hif1α* mRNA and protein levels in diabetes-exposed mouse embryos^16, 41^. Although the regulation of many of the cellular functions of HIF1α has been described to operate at the protein level, increasing evidence indicates that transcriptional control of its expression plays an important role^58^. In this regard, *Hif1α* expression increases in response to glucose via regulation at the promoter level^59^. During diabetic pregnancy both hypoxic and oxidative stress occur concomitantly^60^ and stimulate HIF1α function^61^. The increased expression of *Hif1α* in embryos developing during diabetic pregnancies provides a defence mechanism to protect them from the toxic effects of glucose^16, 41^. Although ALX3 does not appear to regulate *Hif1α* directly, our data support the notion that the stimulated increase of ALX3 in response to elevated glucose levels plays an important role to coordinate the protective response of the embryo to maternal diabetes because *Alx3*-deficiency significantly impairs the enhanced expression of embryonic *Hif1α* and oxidative stress detoxifying genes during diabetic pregnancy.

Maternal hyperglycaemia alters gene expression patterns in the embryo, and this may play an important role in the pathogenesis of congenital defects during diabetic pregnancy^10, 16, 19, 62, 63^. *Alx3* deficiency in embryos exposed to high glucose concentrations *in utero* results in decreased expression of *Ap2*, *Bmp4* and *Pdgfrα*, chosen as representative examples of genes whose deficiencies cause congenital defects that are similar to some of the craniofacial defects found in *Alx3*-deficient embryos. AP2 is expressed in mesenchyme cells that are important craniofacial and neural development^49, 64^. BMP4 and PDGFRα are signalling molecules that control different aspects of craniofacial mesenchyme differentiation^51, 65^. Importantly, we only detected significant changes in the expression of *Ap2*, *Pdgfrα* and *Bmp4* when both *Alx3* deficiency and maternal diabetes were present together. Although many other genes are likely to be affected by maternal diabetes^16, 19, 62, 63^, these changes represent indications that *Alx3* is important for the protection of transcriptional and cell signalling programs that are critical for embryonic development from the noxious effects of hyperglycaemia. Interestingly, *Gcn5* expression was not affected by *Alx3* deficiency even in the presence of maternal diabetes. Since *Gcn5* deficiency results in embryonic neural tube defects without affecting mesenchyme development^53^, this observation suggest that the impact of maternal diabetes is greater in *Alx3*-deficient mesenchyme cells than in other cell types in which *Alx3* is not normally expressed.

We looked for evidence of altered gene expression in *Alx3*-deficient embryos from diabetic pregnancies showing no major malformations to avoid the detection of changes that could be attributable to alterations in cellular composition associated to dysmorphogenesis. This is important because incomplete penetrance of congenital malformations constitutes a common feature of birth defects induced by maternal diabetes, so that a proportion of embryos develop normally despite the detection of significant changes in gene expression^16, 63^. This may be due to increased variability in gene expression levels induced by maternal diabetes, so that defects would only appear when expression of certain genes fall below a given threshold^63^. In this context, *Alx3* deficiency would render the embryos prone to the appearance of overt developmental alterations by increasing permissively the magnitude of these changes. Therefore, changes in the expression of genes observed in *Alx3*-deficient embryos from diabetic mothers do not necessarily identify direct targets of regulation by ALX3. Instead, these changes reflect a role for ALX3 as a protective genetic buffer to temper the effects of hyperglycaemia on gene expression levels during a critical period of development.

In conclusion, our data reveal the existence of a novel defence mechanism to prevent congenital malformations due to oxidative stress during diabetic pregnancies. In this mechanism, *Alx3* participates as a glucose-responsive gene required for the activation of *Foxo1* and the subsequent stimulation of oxidative stress-scavenging genes. In humans, recessive *ALX3* mutations have been shown to cause congenital craniofacial alterations^34^. Thus, taken together in the context of the present study, loss of function mutations in *Alx3* emerge as putative determinants for increased risk of congenital craniofacial malformations associated with diabetes during pregnancy.

## Methods

### Mice

*Alx3*-deficient mice^33^ were maintained in a C57BL/6 J × FVB/N background by heterozygote intercrosses and were genotyped using PCR as described^35^. Experimental protocols involving mice were approved by the institutional bioethics committees on research animal care (IIBM Committee on Human and Animal Experimentation and CSIC Ethics Committee), and are in accordance with the requirements of Spanish (RD 53/2013) and European Union (63/2010/EU) legislation.

### Induction of insulin-dependent diabetes and pregnancy

Insulin-dependent diabetes and pregnancy in mice were induced largely as described^40^. Briefly, 8–10 week old wild type or *Alx3* ^−/−^ female mice were treated with streptozotocin (Sigma-Aldrich, Madrid, Spain; 75 mg/kg, i.p.) for four consecutive days to destroy pancreatic islets. The rise in blood glucose levels after the injections was monitored with a glucometer (Glucotrend Soft Test System; Boehringer Ingelheim, Mannheim, Germany) using tail blood, and hyperglycaemia was controlled by subcutaneous implantation of sustained-release insulin pellets (LinShin, Toronto, Ontario, Canada). Stable recovery of blood glucose levels after pellet implantation was monitored regularly. After confirming that blood glucose levels were stable for 2–3 weeks, females were mated with non-diabetic wild type or *Alx3* ^−/−^ male mice. Noon on the day in which a vaginal plug was found was considered to be day 0.5 of gestation. Blood glucose levels were monitored from this time on every three days. On gestation day 10.5, pregnant mice were killed by cervical dislocation and the embryos were extracted for analyses. As a control, a different group of streptozotocin-treated females received insulin pellets but were not mated to males. These non-pregnant females did not develop diabetes during a similar period of time. Islet destruction by streptozotocin was confirmed in histological sections of the pancreases processed for insulin immunofluorescence (Supplementary Fig. S3).

### Glucose tolerance test and insulin determinations

Glucose levels were measured with a glucometer using blood obtained by puncture of the tip of the tail after an overnight fasting period. After measuring baseline glucose levels, mice were injected with glucose (2 g/kg, i.p.), and blood was tested 15, 60 and 120 minutes after the injection. Glucose tolerance tests were carried out both before pregnancy and 8.5 days after coitus. Fasting (4 hours) serum insulin was measured before pregnancy and 10.5 days post coitus using an ELISA kit (Crystal Chem, Downers Grove, IL, USA).

### Immunohistochemistry

Cryostat sections (10 μm) of embryos were processed for anti-nitrotyrosine immunohistochemistry using a specific antibody (Millipore, Billerica, MA, USA; Ref. 06-284, 1:1000 dilution). Immunodetection was carried out with a secondary biotinylated goat anti-rabbit antiserum (Bio-Rad) using immunoperoxidase staining. The expression of nitrotyrosine was scored by quantifying the number of positive cells per region of interest from digital images using NIH ImageJ software. In all sections regions of interest located both on the left and right sides of the embryo were scored independently. Counterstaining was carried out with haematoxylin. Immunofluorescence on pancreas sections with an insulin antibody (Ab7842, Abcam; 1/100 dilution) was performed as described^35^.

### Mouse embryonic mesenchyme cells

Primary MEM cells were obtained from the forehead mesenchyme of embryos taken from time-pregnant females at gestation day 10.5 as described^31^. This location and gestation time were chosen to maximize the number of cells expressing *Alx3* ^30, 32^. Briefly, the first branchial arch and the most rostroventral part of the forehead mesenchyme of each embryo was dissected and pieces from several embryos of the same genotype were pooled. Once dispersed, cells were incubated in standard DMEM containing 10% FBS and split a maximum of three times. In one set of experiments, primary MEM cells were incubated in medium containing 2.8 mM glucose overnight. After this, the medium was replaced with fresh medium containing either 2.8 mM, 16 mM or 30 mM glucose, and the incubation proceeded for another 24 hours until cells were harvested. In a different set, cells were incubated in DMEM containing 0.5% FBS and 0.5% BSA, and insulin (Human recombinant, Sigma I2643) was added to a concentration of 100 nM overnight.

### Intracellular ROS measurement by fluorescence-activated cell sorting

ROS levels in primary MEM cells cultured in different concentrations of glucose or treated with t-BOOH (Sigma-Aldrich) were determined after incubation for 30 minutes in the dark with the fluorescent probes 5-chloromethyl-2′,7′-dichlorodihydrofluorescein diacetate acetyl ester (CM-H_2_DCFDA, 0.5 μM; Invitrogen) or dihydroethidium (DHE, 2 μM; Invitrogen). CM-H_2_DCFDA is a general ROS indicator that detects primarily hydrogen peroxide, whereas DHE specifically detects the superoxide anion^66^. Cell viability was assessed by measuring the plasma membrane permeability to 4′,6-Diamidino-2-phenylindole dihydrochloride (DAPI; 1 μg/ml, ThermoFisher Scientific/Molecular Probes, Waltham, MA, USA) or to propidium iodide (1 μg/ml, Sigma-Aldrich, St. Louis, MO)^67^. Fluorescence (10,000 cells/sample) was measured by flow cytometry using a FACS Aria I flow cytometer (Becton–Dickinson, Franklin Lakes, NJ, USA). All measurements were carried out in duplicate.

### Quantitative RT-PCR

Total RNA from MEM cells or from individual embryos was extracted using the Illustra RNAspin kit (GE Healthcare Europe, Barcelona, Spain). Embryos showing major malformations were not used for mRNA determinations to avoid artefacts arising directly from embryonic dysmorphogenesis^16^. Determinations were carried out in the Core Facilities of the Genomics Unit at the Scientific Park of Madrid (http://fpcm.es). Quantitative PCR for *Alx3* was performed with TaqMan Assay-on-Demand primers and the Taqman Universal PCR Master Mix, No AmpErase UNG (Applied Biosystems, Alcobendas, Madrid, Spain). In other cases, SYBR Green detection was used with Power SYBR Green PCR Master Mix (Applied Biosystems) and the primers indicated in Supplementary Table S2. PCR reactions were performed in triplicate and values were normalized to *Gapdh* mRNA levels using the double delta Ct method. Stability of *Gapdh* as a reference for normalization was confirmed using geNorm^68^, and results are shown in Supplementary Fig. S4.

### Western blot

Western blots from primary MEM cell lysates were performed as described^31^. Antibodies for ALX3 (1:5000 dilution)^31^, FOXO1 (L27, 1:1000 dilution; Cell Signalling Technology, Beverly, MA, USA) and β-actin (AC-15, 1:10000 dilution; Sigma-Aldrich) were used for detection. Where indicated, films were scanned and densitometry measurements of bands were performed using ImageJ software (http://rsbweb.nih.gov/ij/).

### Chromatin immunoprecipitation assays

Chromatin immunoprecipitation assays were performed as described^69^. Quantitative PCR was performed using oligonucleotide primers that amplify a fragment of the mouse *Foxo1* gene spanning nucleotides −880 to −751 relative to the transcription initiation site. Their sequence is as follows: Forward, 5′-TGCGACTTCAACACCTCATC-3′; reverse, 5′-CGGTGTGGTGGCTAAAGAGT-3′. SYBR Green detection was used with Power SYBR Green PCR Master Mix (Applied Biosystems). Data were calculated as fold enrichment relative to input.

### Electrophoretic mobility shift assays

Nuclear protein extracts from primary MEM cells were prepared and electrophoretic mobility shift assays were performed as described^69^. The sequence of the oligonucleotide from the mouse *Foxo1* promoter is as follows (sense strand): 5′-GATCCAGTTAAACTTAATTAGGGGTAATAAAGTGA-3′. For supershift experiments, nuclear extracts were preincubated with an ALX3 antibody (ab64985, Abcam, Cambridge, MA, USA) before the addition of the labelled probe.

### Plasmids and transfections

A segment of the mouse *Foxo1* gene promoter spanning nucleotides −880 to −751 relative to the transcription initiation site was amplified by PCR using the following primers: Forward, 5′-CAGGATCCTGCGACTTCAACACCTCATC-3′; and reverse, 5′-CAAAGCTTCGGTGTGGTGGCTAAAGAGT-3′. The resulting fragment was cloned into the luciferase reporter plasmid pT81Luc^70^ to yield Foxo1T81Luc. The expression vector pcDNA3-Alx3 has been described elsewhere^45^.

HeLa cells or primary MEM cells prepared from *Alx3*-deficient embryos were seeded into 24-well plates at a density of 7 × 10^4^ cells per well and incubated overnight. Cells were transfected for 4 hours with Lipofectamine (Invitrogen), using 500 ng of luciferase reporter plasmid, 50 ng of pRL-TK-*Renilla* and pcDNA3-Alx3 or control pcDNA3 plasmids. Luciferase and *Renilla* activities were measured using a commercial Dual Luciferase Assay kit (Promega, Madrid, Spain) 48 hours after transfection. The reporter plasmid RSV-Luc was used as an independent standard for normalization, and efficiencies were corrected by using the *Renilla* luciferase assay system (Promega). All transfections were carried out in duplicates.

### Statistical analysis

The incidence of malformations was analysed by the Fisher exact test. The presence of synergism between hyperglycaemia and *Alx3* deficiency was tested by comparing observed and expected malformation frequencies using χ^2^ test. All other data are expressed as mean ± s.e.m., and were analysed by Student’s *t*-test or two-way ANOVA followed by Bonferroni transformation, using Package GraphPad Prism 6 software.

## Electronic supplementary material

Supplementary Figures and Tables
