# Supplementary material for: Embryonic defence mechanisms against glucose-dependent oxidative stress require enhanced expression of Alx3 to prevent malformations during diabetic pregnancy
Source: Sci Rep. 2017 Mar 24;7:389. doi: 10.1038/s41598-017-00334-1 (PMC5428206; doi:10.1038/s41598-017-00334-1)
Supplement: Supplementary file 1 — Supplementary Figures and Tables [file 41598_2017_334_MOESM1_ESM.pdf]

Supplementary information: Embryonic defence mechanisms against glucose-dependent oxidative stress require enhanced expression of *Alx3* to prevent malformations during diabetic pregnancy. García-Sanz, P., Mirasierra, M., Moratalla, R. and Vallejo, M.

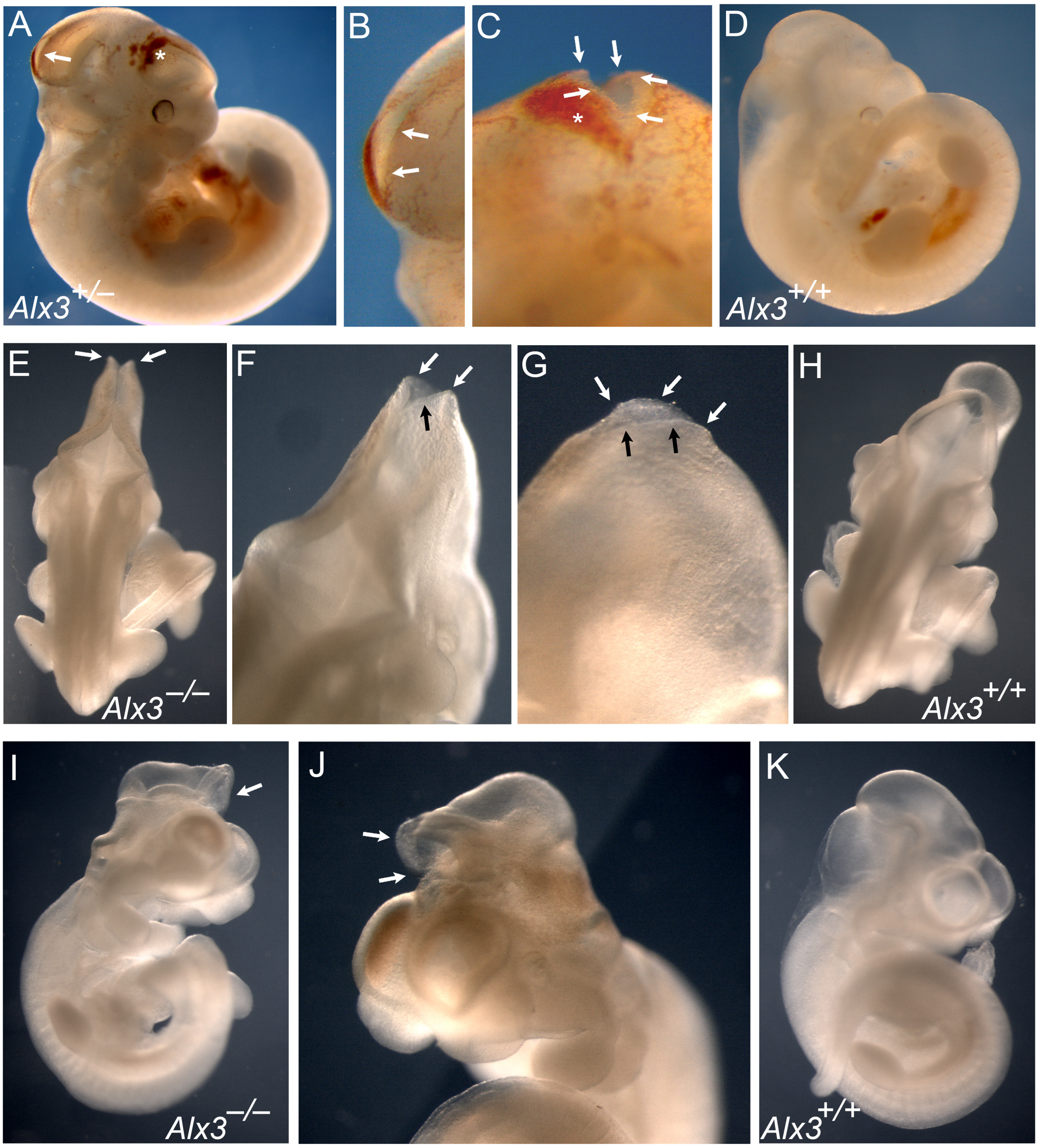

Supplementary Figure S1. Examples neural tube closure defects observed in heterozygote or homozygote *Alx3*-mutant embryos from non-diabetic pregnancies examined after 10.5 days of gestation. A-C) $Alx3^{+/-}$ embryo with incomplete closure at the level of the mesencephalon. Abnormally open neural folds are indicated by arrows. B and C, Higher magnification images showing a lateral (B) or a dorsal (C) view. The asterisk denotes the abnormal presence of haemorrhages. D) Normal littermate shown for comparison. E-G) $Alx3^{-/-}$ embryo with incomplete mesencephalic closure. Arrows indicate abnormally open neural folds. E, Dorsal view; F and G, Dorsolateral and lateral views of the same embryo, respectively, shown at higher magnification. H) Dorsal view of a normal littermate with the neural tube properly closed shown for comparison. I and J) A different $Alx3^{-/-}$ embryo with a defect in neural tube closure at the level of the prosencephalic-mesencephalic boundary (arrows). J, Ventrolateral view of the same embryo shown at higher magnification. K) A normal littermate shown for comparison. A detailed characterization of neural tube closure and craniofacial midline defects in *Alx3*-deficient embryos has been reported previously by our group (ref. 33).

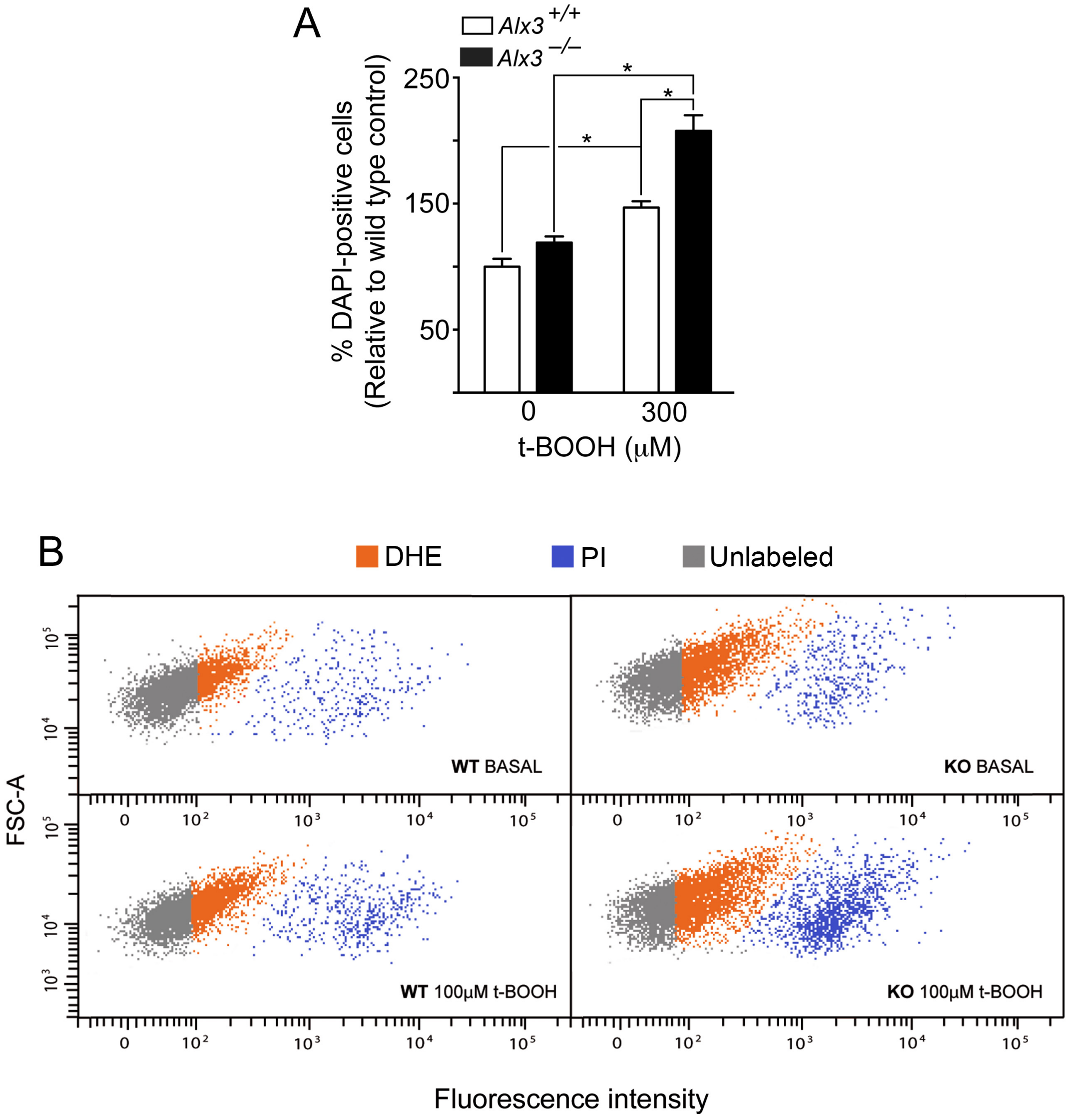

Supplementary Figure S2. FACS experiments to determine the viability of primary MEM cells subjected to oxidative stress in response to t-BOOH treatment. A) Response of control or *Alx3*-deficient primary MEM cells to 300 µM t-BOOH treatment. The relative numbers of non viable cells identified by DAPI staining is represented (n = 3 in each cell group. **P*< 0.001, ANOVA followed by Bonferroni test. All values represent mean $\pm$ s.e.m. B) Dot plots of FACS analysis before (upper panels) and after (lower panels) treatment of cells with 100 µM t-BOOH. Wild type (left panels) or *Alx3*-deficient cells (right panel) were labelled with DHE (orange dots) and propidium iodide (PI, blue dots) and analysed by flow cytometry. Grey dots represent unlabelled viable cells.

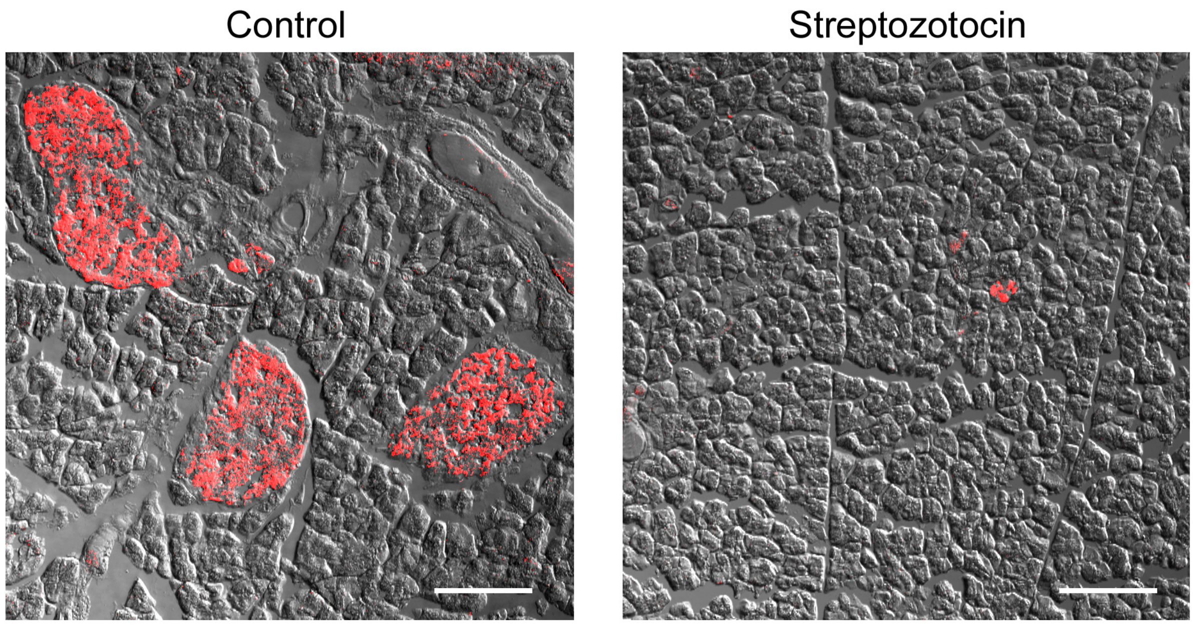

Supplementary Figure S3. Pancreatic islet destruction by streptozotocin. Shown are representative examples of sections from the pancreases of mice which were untreated (Control)) or treated with streptozotocin. Histological sections were processed for insulin immunofluorescence and analysed by confocal microscopy. The immunofluorescent image (red) was superimposed on an image of the same field of view obtained by Nomarski differential interference contrast microscopy to visualize the exocrine pancreas on the section. Note the almost total absence of insulin immunofluorescence in sections from streptozotocin treated animals. The scale bar represents 100 μm.

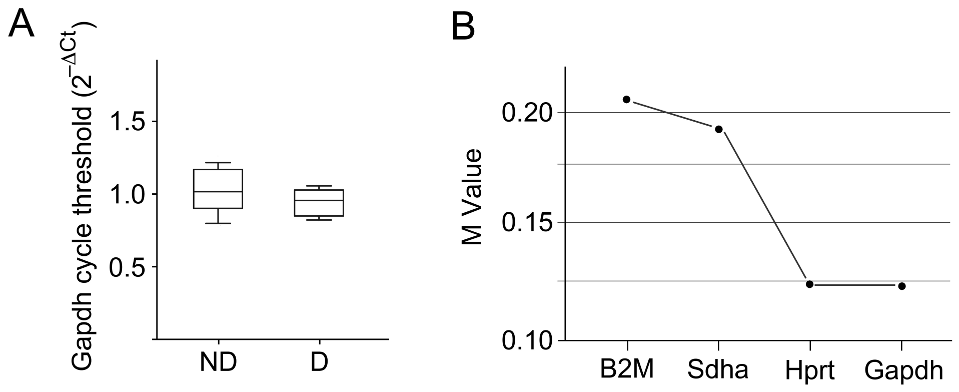

Supplementary Figure S4. Confirmation of the stability of the expression of *Gapdh* for use as a reference in RT-qPCR experiments. A) Expression levels of *Gapdh* in samples of mouse embryos of 10.5 days of gestational age, obtained from non-diabetic (ND) or diabetic (D) mothers. Data were calculated from real-time PCR cycle threshold numbers (Ct values) and are represented as box-and-whisker plots (10-90 percentiles) showing median values as well as first and third quartiles. No statistically significant differences were found between groups (n = 24 per group). **B**) GeNorm analyses for beta-2 microglobulin (B2M), succinate dehydrogenase A subunit (Sdha), hypoxanthine-guanine phosphoribosyltransferase (Hprt) and glyceraldehyde 3-phosphate dehydrogenase (Gapdh), four commonly used reference genes in RT-qPCR analyses. Note that the M stability value for *Gapdh* is well below 0.5 considered acceptable for homogeneous samples.

Supplementary Figure S5: Expanded blots and EMSA shown in main Figure 4

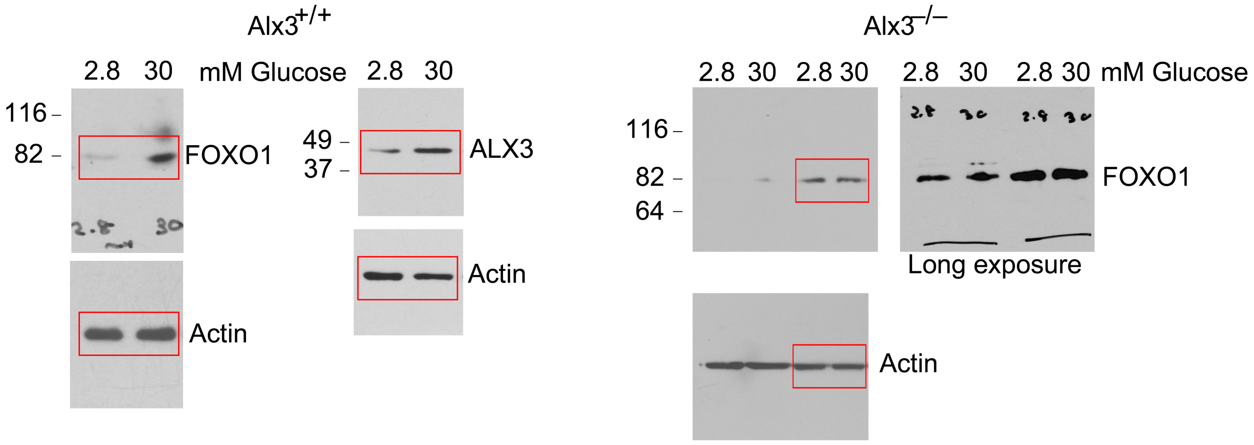

Expanded data Figure 4B and K

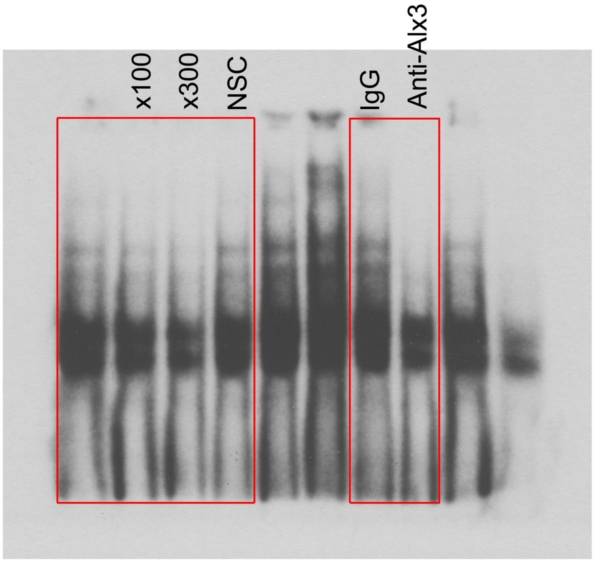

Expanded data Figure 4F

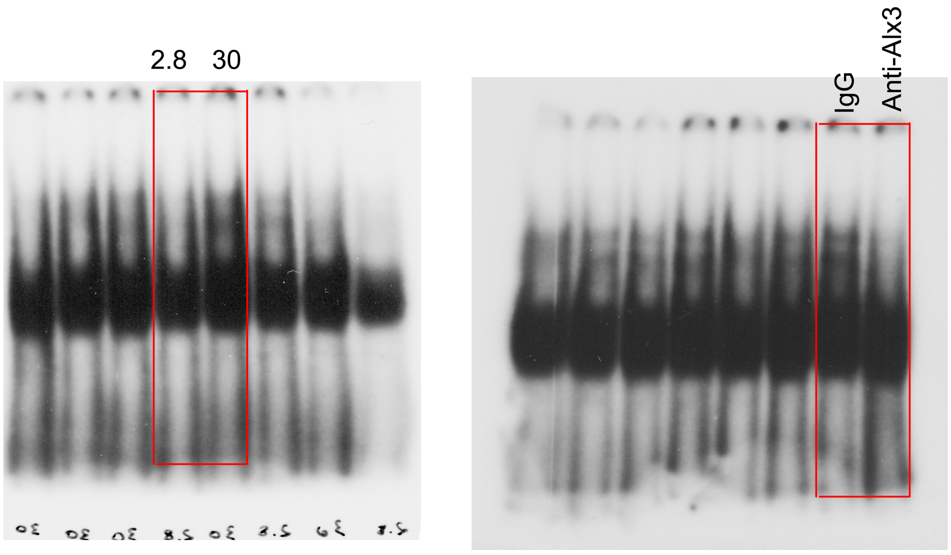

Expanded data Figure 4J

Supplementary Table S1. Basal serum insulin levels (ng/ml) in non-pregnant or pregnant (10.5 days of gestation) mice.

	**Alx3 $^{+/+}$**	**Alx3 $^{-/-}$**
Non-pregnant	0.72 $\pm$ 0.21	0.81 $\pm$ 0.17
Pregnant	0.85 $\pm$ 0.18	0.79 $\pm$ 0.24

n = 6 in each group.

Supplementary Table S2. Oligonucleotide primers used in quantitative RT-PCR experiments

	Forward	Reverse
AP2	GTGTCAGAGATGCTGCGGTA	TGAGGATGGTGTCCACGTA
BMP4	CGAGCCAACACTGTGAGGAGT	AGGTTGAAGAGGAAACGAAAAGC
Catalase	GCAGATACCTGTGAACTGTC	GTAGAATGTCCGCACCTGAG
Foxo1	TATTGAGCGCTTGGACTGTG	TGGACTGCTCCTCAGTTCCT
Foxo4	TGTAACAGGTCCTCGGAAGG	CTGTGCAAGGACAGGTTGTG
Gnc5	AGTTGTGCCGTAGCTGTGAG	TGGTGTCTGTGTCCTCTTCC
Gpx1	CCTCAAGTACGTCCGACCTG	CAATGTCGTTGCGGCACACC
Hif1α	CAGTACAGGATGCTTGCCAAAA	ATACCACTTACAACATAATTCACACACACA
iNOS	CTCACTGGGACAGCACAGAA	TGGTCAAACTCTTGGGGTTC
MnSOD	GCACATTAACGCGCAGATCA	AGCCTCCAGCAACTCTCCTT
nNOS	CCTGTGGGAGTCGTCTTGGC	GTGGTCTCCAGGTGTGTAGTAAAGC
Nrf2	TCACACGAGATGAGCTTAGGGCAA,	TACAGTTCTGGGCGGCGACTTTAT
PDGFRα	GACGAGTGTCCTTCGCCAAAGTG	CAAAATCCGACCAAGCACGAGG
